# Diffusion MRI approaches for investigating microstructural complexity in a rat model of traumatic brain injury

**DOI:** 10.1038/s41598-023-29010-3

**Published:** 2023-02-08

**Authors:** Karthik Chary, Eppu Manninen, Jade Claessens, Alonso Ramirez-Manzanares, Olli Gröhn, Alejandra Sierra

**Affiliations:** 1 A.I. Virtanen Institute for Molecular Sciences, University of Eastern Finland, P.O. Box 1627, 70211 Neulaniementie 2, Kuopio, Finland; 2 Department of Radiology, University of Cambridge, Cambridge, UK; 3 Department of Computer Science, Centro de Investigación en Matemáticas, Guanajuato, Mexico

**Keywords:** Neuroscience, Brain, Experimental models of disease, Preclinical research, Brain injuries, Neurodegenerative diseases

## Abstract

Our study explores the potential of conventional and advanced diffusion MRI techniques including diffusion tensor imaging (DTI), and single-shell 3-tissue constrained spherical deconvolution (SS3T-CSD) to investigate complex microstructural changes following severe traumatic brain injury in rats at a chronic phase. Rat brains after sham-operation or lateral fluid percussion (LFP) injury were scanned ex vivo in a 9.4 T scanner. Our region-of-interest-based approach of tensor-, and SS3T-CSD derived fixel-, 3-tissue signal fraction maps were sensitive to changes in both white matter (WM) and grey matter (GM) areas. Tensor-based measures, such as fractional anisotropy (FA) and radial diffusivity (RD), detected more changes in WM and GM areas as compared to fixel-based measures including apparent fiber density (AFD), peak FOD amplitude and primary fiber bundle density, while 3-tissue signal fraction maps revealed distinct changes in WM, GM, and phosphate-buffered saline (PBS) fractions highlighting the complex tissue microstructural alterations post-trauma. Track-weighted imaging demonstrated changes in track morphology including reduced curvature and average pathlength distal from the primary lesion in severe TBI rats. In histological analysis, changes in the diffusion MRI measures could be associated to decreased myelin density, loss of myelinated axons, and increased cellularity, revealing progressive microstructural alterations in these brain areas five months after injury. Overall, this study highlights the use of combined conventional and advanced diffusion MRI measures to obtain more precise insights into the complex tissue microstructural alterations in chronic phase of severe brain injury.

## Introduction

Traumatic brain injury (TBI) is a major cause of disability, morbidity, and deaths worldwide^1^. The use of neuroimaging techniques for clinical assessment of TBI patients is common practice with computed tomography being the method of choice in the acute phase, to evaluate hemorrhage or skull fractures sustained post-injury. In the more subacute and chronic phases post-injury, magnetic resonance imaging (MRI) techniques have yielded more in-depth knowledge of brain tissue damage^2–4^.

The myriad of secondary tissue changes following the initial blow renders TBI to be a very complex pathology^5–7^. These secondary tissue changes are associated with a broad spectrum of symptoms and disabilities such as motor impairments, cognitive and/or mood disorders^8–11^. At the tissue level, neurodegeneration, inflammation, axonal injury, demyelination, iron accumulation or calcifications can persevere for months or several years post injury^6,12–14^. However, conventional MRI approaches such as *T*_*1*_, *T*_*2*_ or *T*_2_* are limited in sensitivity and specificity in probing underlying microstructural alterations related to secondary damage^15–17^. Improved non-invasive detection of TBI-related pathological tissue changes can lead to a significant improvement on the diagnosis and early treatment, and therefore quality of life of TBI patients.

By probing translational molecular motion, diffusion MRI-based approaches are sensitive to changes in water diffusion due to pathology-related alterations in tissue microstructure. More specifically, diffusion tensor imaging (DTI)^18^ has demonstrated sensitivity in revealing microstructural tissue changes post-TBI both in humans^17,19–21^ and experimental animals^22–25^. Even though single tensor-derived metrics can detect microstructural changes following TBI, more advanced approaches may comprehensively describe the complex changes of underlying pathology overall following TBI^26,27^. Constrained spherical deconvolution (CSD) has been developed to provide a more precise assessment of the microstructural environment in a voxel^28,29^. Previous studies have shown CSD-based frameworks including fixel-based analysis (FBA) and track-weighted imaging (TWI) to be more precise to detect axonal damage, and demyelination, following mild^30,31^ and severe TB^30,32^ in rats in vivo. Furthermore, by estimating tissue-specific response functions, the single-shell 3-tissue variant of CSD (SS3T-CSD)^32,33^, has demonstrated to provide stable and reliable evaluation of microstructure composition in healthy tissues^34^. In pathology, SS3T-CSD has exhibited greater precision to assess axonal injury microstructurally^35,36^ and microstructural heterogeneity in white matter hyperintensities^36^ in Alzheimer’s disease, and free-water estimation^37^ and correction^38^ in other neurological disorders. Moreover, SS3T-CSD has also enabled more reliable estimation of white matter microstructure both adjacent to and inside brain tumours^39^, and compositional analysis^40^ to further probe the diffusion signal characteristics associated with tissue-specific microstructural heterogeneity of white matter lesions in stroke^41^ and Alzheimer’s disease^35^. Although these studies provide more precision about the tissue microstructure in the pathological brain, SS3T-CSD is a new technique, and the single-shell approach would benefit from studies with histological corroboration to evaluate its potential and limitations in assessing pathological tissue.

Therefore, in this study, we aim to evaluate the complex tissue-specific microstructural changes following severe brain injury using DTI-, and SS3T-CSD derived fixel, 3-tissue signal fraction maps, and TWI. For this, we performed a region-of-interest (ROI)-based analysis on tensor-, fixel-based scalar maps, combined with compositional analysis of 3-tissue signal fraction maps in sham-operated and severe TBI rat brains ex vivo five months after injury. In addition, we segregated the thalamocortical tract using targeted tractography for assessing changes in track morphology following severe TBI. To corroborate MRI findings, histological validation was performed using stained sections for myelin and Nissl in selected brain regions. Overall, this study highlights the potential of both conventional DTI and advanced SS3T-CSD methodologies to detect the microstructural complexity at a chronic phase following severe brain injury.

## Methods

### Animal model

Experimental TBI was induced in male Sprague-Dawley rats (n = 12; 10 weeks old, 300–350 g, Harlan Netherlands B.V., Horst, The Netherlands) by lateral fluid percussion (LFP) injury as described previously^42^. In brief, rats were anesthetized with a single i.p. injection (6 ml/kg) of a mixture containing sodium pentobarbital (Mebunat, Orion Oy, Finland; 60 mg/kg), magnesium sulphate (127.2 mg/kg), propylene glycol (39.5%), and absolute ethanol (10%). Then, a craniectomy (∅ = 5 mm) was performed between bregma and lambda on the left skull convexity, whose center was approximately at − 4.50 mm posterior to the bregma and lateral edge of the craniectomy was adjacent to the left lateral ridge. A fluid percussion device (AmScien Instruments, Richmond, VA, USA) was then used to induce an LFP injury to the exposed dura using a transient fluid pulse (21–23 ms) to induce a severe injury (3.13 ± 0.09 atm). After the injury, we checked that the dura remained intact. Sham-operated rats (n = 8) were subjected to the same operational procedures except for the impact. The mortality rate in the TBI group was 42% (n = 5). One sham-operated rat died during the procedure under anesthesia (12.5%; n = 1).

Following the operation, the animals were transferred to the animal facility and housed in individual cages maintained under a 12 h light/12 h dark cycle (lights on 07:00 a.m., temperature 22 ± 1 °C, humidity 50–60%) with free access to food and water. All animal procedures were carried out under licenses that have been approved by the Animal Ethics Committee of the Provincial Government of Southern Finland and in accordance with the guidelines of the European Community Council Directives 86/609/EREC. This study followed the recommendation in the ARRIVE guidelines.

### Tissue preparation

At a chronic phase, five months after the operation, all the rats (n = 13) were deeply anesthetized under 5% isoflurane in 30%/70% O_2_/N_2_ gas mixture, and transcardially perfused with saline for 2 min (30 ml/min) followed by 4% paraformaldehyde in 0.1 M phosphate buffer, pH 7.4 (30 ml/min) for 25 min. After perfusion, the brains were removed from the skull, post-fixed in a solution of 4% paraformaldehyde for 4 h, and finally washed in 0.9% NaCl solution for 12 h before MRI. The brains were then transferred to a solution of 0.1 M phosphate-buffered saline (PBS) containing 1 mM gadolinium diethylenetriamine penta-acetic acid (Gd-DOTA, Dotarem®, Guerbet, Paris, France) for at least 72 h. Prior to imaging, the brains were placed tightly inside a polyethylene tube filled with perfluoro polyether (Galden HS240, Vacuumservice Oy, Helsinki, Finland) to prevent tissue drying and to effectively suppress the background signal.

### Ex vivo MRI acquisition

Data were acquired in a 9.4 T (400 MHz) 89-mm-vertical bore magnet (Oxford Instruments PLC, Abingdon, UK) using a quadrature volume RF coil (∅ = 20 mm; Rapid Biomedical GmbH, Rimpar, Germany) for signal transmission and reception. A 3D diffusion spin-echo-echo-planar-imaging (SE-EPI) sequence with triple reference was used with TR/TE = 800/35 ms, echo spacing = 0.584 ms, number of shots = 4, bandwidth = 250 kHz, number of averages = 2, FOV (read x phase x phase2) = 16.2 × 12.0 × 14.1 mm^3^, matrix size (read x phase x phase2) = 108 × 80 × 94, spatial resolution = 150 × 150 × 150 μm^3^, number of images with diffusion weighting = 60, b-value = 3019.7 s/mm^2^, number of images with minimal diffusion weighting = 1, gradient amplitude = 350 mT/m, gradient duration (δ)/separation (Δ) = 6/11.50 ms, acquisition time = 20 h 43 min. The triple reference scheme is the manufacturers implementation that requires each diffusion MRI image to be acquired with a reversed readout gradient polarity to reduce Nyquist ghosting artifacts observed in EPI.

### Image processing

K-space data were processed with in-house codes using Matlab (R2017a, MathWorks, Natick, Massachusetts, USA) to reconstruct diffusion weighted images. All processing steps were performed using MRtrix3^43^ and using MRtrix3 scripts interfaced with external software packages. Initially, data quality was assessed by calculating signal-to-noise ratios (SNR) over the whole brain, and subsequently subjected to denoising^44^, Gibbs unringing^45^, and eddy current correction^46^. Data quality was further assessed by EDDY’s QC tool^47^, and data detected as outliers for motion (n(sham) = 1) were removed from subsequent analysis. The mean SNR for the diffusion volumes was 28.50 + /−6.26. Additionally, contrast-to-noise ratios (CNRs) derived from EDDY’s QC tool within each group were also checked for outliers based on the inter-quartile range and were subsequently removed (n(sham) = 1, n(TBI) = 1). Although, a visual inspection found no gross movement of the rat brains and none of the diffusion volumes were found to be unusable, the susceptibility and eddy current related off-resonance effects can be subtle and difficult to visualize across the entire dataset^47^, which can affect overall data quality, and subsequent statistical analyses considering the small sample size, warranting the exclusion of datasets detected as outliers. The final dataset contained 10 rats (n(sham) = 4, n(TBI) = 6). Finally, we performed bootstrap aggregating in Matlab by removing 10 diffusion volumes from each animal and estimated all the diffusion metrics.

### Fixel-based analysis

Tissue specific response functions for single-fiber WM, GM and PBS were estimated in an unsupervised method^48^ from each sham-operated animal and combined to create a group averaged response function. This ensured the estimated FODs and subsequent interpretation of the CSD metrics were unbiased from pathology. As the brains were immersed in PBS before scans, we use the notation PBS instead of CSF as the 3rd tissue type as reported previously^49^. Processed images were upsampled by a factor of two to increase anatomical contrast^50^, to an isotropic voxel size of 75 μm^3^. SS3T-CSD^48^ was performed from the distinct WM, GM, and PBS group averaged response functions to estimate WM-like fiber orientation distributions (FOD), as well as GM-like and PBS-like compartments voxel-wise using MRtrix3Tissue (https://3Tissue.github.io). A spherical harmonic order (lmax = 8) was chosen based on the number of unique diffusion directions in our dataset. The FOD’s were corrected for intensity in-homogeneities using multi-tissue informed intensity normalization.

### Fixel-based metrics

Every FOD lobe from the individual rat brain FODs was segmented into discrete fixels by computing the integral of the FOD lobe using a threshold of 0.06^51^. Total voxel-wise AFD scalar maps were computed from the 1st spherical harmonic coefficient of the FOD. The fixel-based sparse-data images were processed with the fixel2voxel command to compute voxel-based metrics of peak amplitude, total dispersion, and complexity^52^. We also decomposed the fixels in each brain area to extract voxel-wise fiber density maps of the primary- (fixel 1), and secondary fiber bundle (fixel 2), respectively^53^. The changes observed in these fixel-based metrics can provide a measure of the damage in the tissue microenvironment. For instance, a decrease/increase in fiber density (FD) of the primary/secondary fiber bundle might be indicative of loss of myelinated axons, accompanied by the increased presence of more complex cellular processes resulting from damage/loss of myelinated axons, such as infiltration of glial and other cell types^53^.

### 3-tissue signal fractions

The absolute signals from the WM-, GM-, and PBS-like compartments were normalized voxel-wise to sum up to one to compute 3-tissue signal fractions T_W_, T_G_, and T_P_^35,41^ (Supplementary Fig. 1). Changes in these 3-tissue signal fractions only represent changes in the diffusion signal characteristics and do not reflect the actual tissue compartment sizes themselves. For instance, increased GM-like signal is rather indicative of increases in diffusion isotropy and diffusivity due to the increased presence of, e.g., glial cells, and not an actual increase in grey matter. Similarly, increased PBS-like signal indicates an increased free-water content in the form of extra-cellular fluid^35,36^.

### Targeted tractography

Tractograms were generated for each animal along the thalamocortical pathway, both ipsi-and contralaterally by using the ventrobasal complex and perilesional cortex as the seed and target ROIs respectively, and a combination of exclusion ROIs to prevent stray streamlines. The following parameters were used: minimum/maximum length = 0.15/10 mm, angle = 45°, number of tracks = 10,000. Streamline tractography was performed 5 times and tractograms from each iteration were concatenated, following which track-weighed measures including average pathlength map^54^, and mean curvature^55^ were computed.

### Tensor-based analysis

Data were corrected for bias field effects using default parameters^56^ and fitted for the tensor model. Subsequently, the following tensor-based metrics were derived; fractional anisotropy (FA), axial diffusivity (AD), radial diffusivity (RD) and mean diffusivity (MD).

### Quantitative analyses

**Figure 1:**
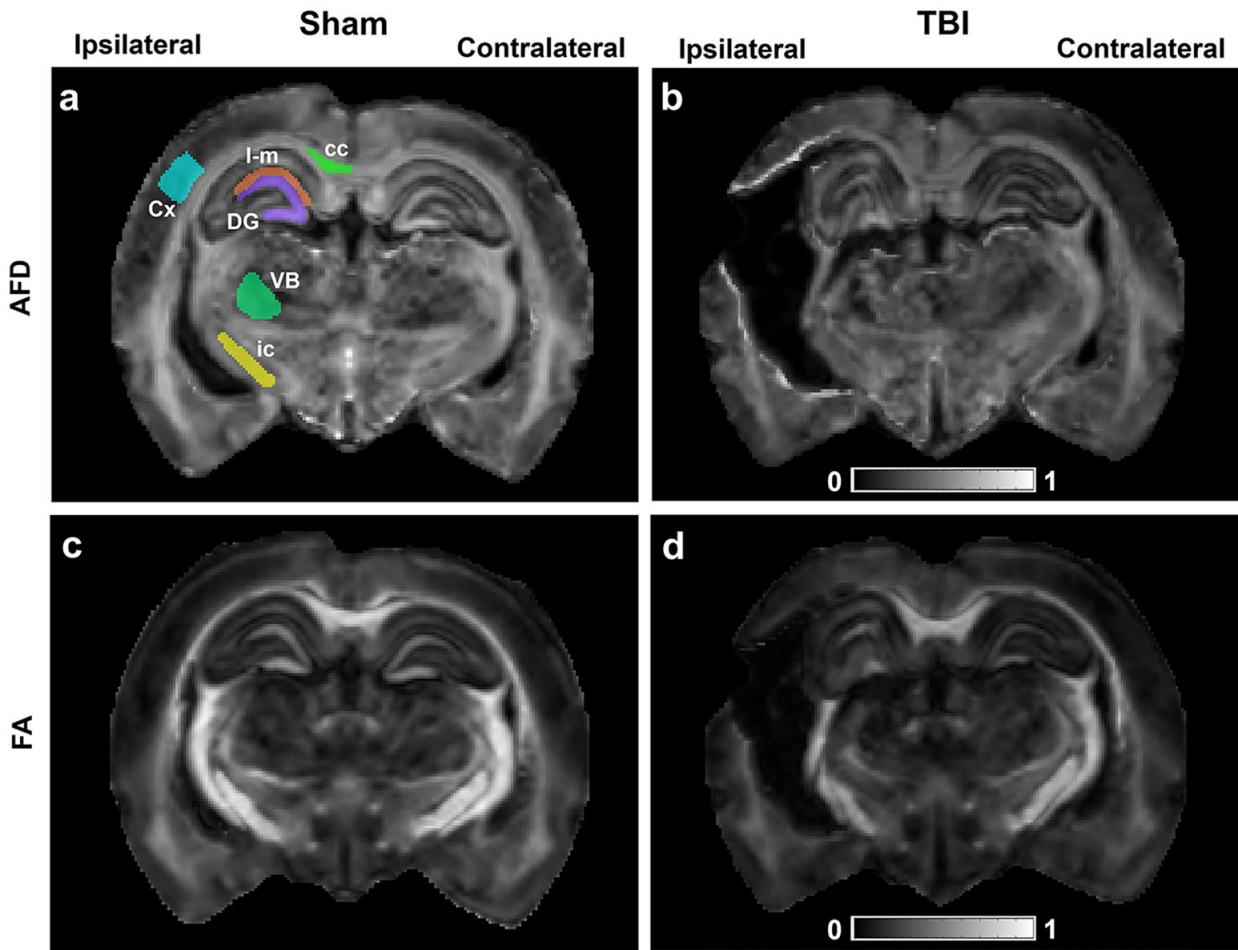
Representative AFD (**a**, **b**) and FA (**c**, **d**) maps from a sham-operated (**a**, **c**) and TBI (**b**, **d**) rat brain. Regions-of-interest were delineated on three consecutive slices at − 3.80 mm from bregma. Abbreviations: AFD, apparent fiber density; cc, corpus callosum; Cx, perilesional cortex; DG, dentate gyrus; FA, fractional anisotropy; ic, internal capsule; l-m, stratum lacunosum-moleculare; VB, ventrobasal complex.

Six ROIs were delineated on the voxel-based AFD maps on both the contralateral and ipsilateral sides using the Aedes software package (https://github.com/mjnissi/aedes). Based on our MRI and histology findings, the epicenter of the lesion, where the damage was most pronounced, appeared at − 3.80 mm from bregma. The location of the epicenter was consistent across animals. ROIs were manually delineated for each animal on three consecutive slices at this level on white and grey matter areas including the corpus callosum, internal capsule, primary somatosensory cortex, ventrobasal complex, dentate gyrus, and stratum lacunosum-moleculare (Fig. 1). These areas were chosen as they demonstrated microstructural alterations using DTI and histology following LFP injury in rats previously^23,24,57^. The delineated ROIs were transferred to tensor- and fixel-based maps to extract their respective mean values.

### Statistical analyses

All data were analysed using Scipy^58^, Statsmodels^59^ (v1.7.1), and Pingouin^60^ (v0.4.0) packages in Python3 (v3.7.4)^61^. Data were first checked for normality using the Shapiro–Wilk test^62^. Differences in tensor- (FA, AD, RD, and MD) and fixel-based metrics (AFD, peak amplitude, dispersion, primary FD, secondary FD, and complexity) between sham-operated and TBI rats were compared using unpaired two-sample t-test, and differences between ipsi- and contralateral brain areas within the same brain using the paired t-test.

For the three-tissue signal fraction maps, a compositional data analysis framework was incorporated as described in^35,41,48^. Briefly, the data were converted into isometric log-ratio transforms^63^ as conventional parametric tests were deemed unfeasible due to the interdependence and boundedness of these maps i.e., *T*_*W*_ + *T*_*G*_ + *T*_*P*_ = 1, and 0 < *Tw*, *T*_*G*_, *T*_*P*_ < 1.

1 $$ilr_{1} = \frac{1}{\sqrt 6 } \times log\frac{{T_{W}^{2} }}{{\left( {T_{P} \times T_{G} } \right)}}$$

2 $$ilr_{2} = \frac{1}{\sqrt 2 } \times log\frac{{T_{P} }}{{\left( {T_{G} } \right)}}$$

The data in *ilr* space were subsequently checked for multivariate outliers using Mahalanobis distances (*q* < *0.01*)^64^, and for normality using the Henze-Zirkler Multivariate Normality Test (*q* < *0.01*)^60,65^. Finally, statistical analyses were performed using MANOVA between sham-operated and TBI rats, and between ipsi- and contralateral hemispheres within the same brain. Pillai’s trace was reported as the test statistic.

All the statistical tests reported in this study were corrected for multiple comparisons using the Benjamini–Hochberg procedure^66^, with the false discovery rate controlled at 0.05 used previously in^67^.

### Histology

After ex vivo imaging, all the brains were washed in 0.9% NaCl for at least 2 h at 4 °C. After this, they were placed in a cryoprotective solution containing 20% glycerol in 0.02 M potassium phosphate-buffered saline (pH = 7.4) for 36 h. Then, the brains were blocked, frozen in dry ice, and preserved at − 70 °C until sectioning. The brains were sectioned in the coronal plane (30 μm, 1-in-5 series) using a sliding microtome. Sections from the first series were stored in 10% formalin while the remaining series were stored in a cryoprotectant tissue-collecting solution (30% ethylene glycol, 25% glycerol in 0.05 M sodium phosphate buffer) at − 20 °C until further processing. The first series of sections was Nissl-stained for thionin to assess the cytoarchitecture, gliosis, and severity of tissue damage in different areas of the brain. The second series of sections was stained with a gold chloride solution for myelin^23^. In brief, the myelin staining was performed on all sections mounted onto gelatin-coated slides and dried at 37˚C. The sections were then incubated in the dark for a period of 5 h in a solution containing gold chloride (HAuCl_4_·3H_2_O; G-4022, Sigma-Aldrich, MO, USA) in 0.02 M sodium phosphate buffer in 0.09% NaCl, pH 7.4. The slides were washed in 0.02 M sodium phosphate buffer in 0.09% NaCl twice for 4 min and placed in a 2.5% sodium thiosulfate solution for 5 min. After the procedure, the sections were again washed three times in phosphate buffer for 10 min each. Finally, the sections were dehydrated through an ascending series of ethanol, cleared in xylene, and cover-slipped with DePeX mounting medium (BDH, Laboratory Supplies, Dorset, UK). Qualitative assessment of the histological sections was done by an experienced neuroscientist (A.S.), and representative cases of the sham-operated and TBI group were selected to corroborate microstructural tissue changes in selected areas. High-resolution photomicrographs of myelin and Nissl-stained sections were acquired using a light microscope (Zeiss Axio Imager 2, White Plains, NY, USA) equipped with a digital camera (Zeiss Axiocam color 506).

## Results

**Figure 2:**
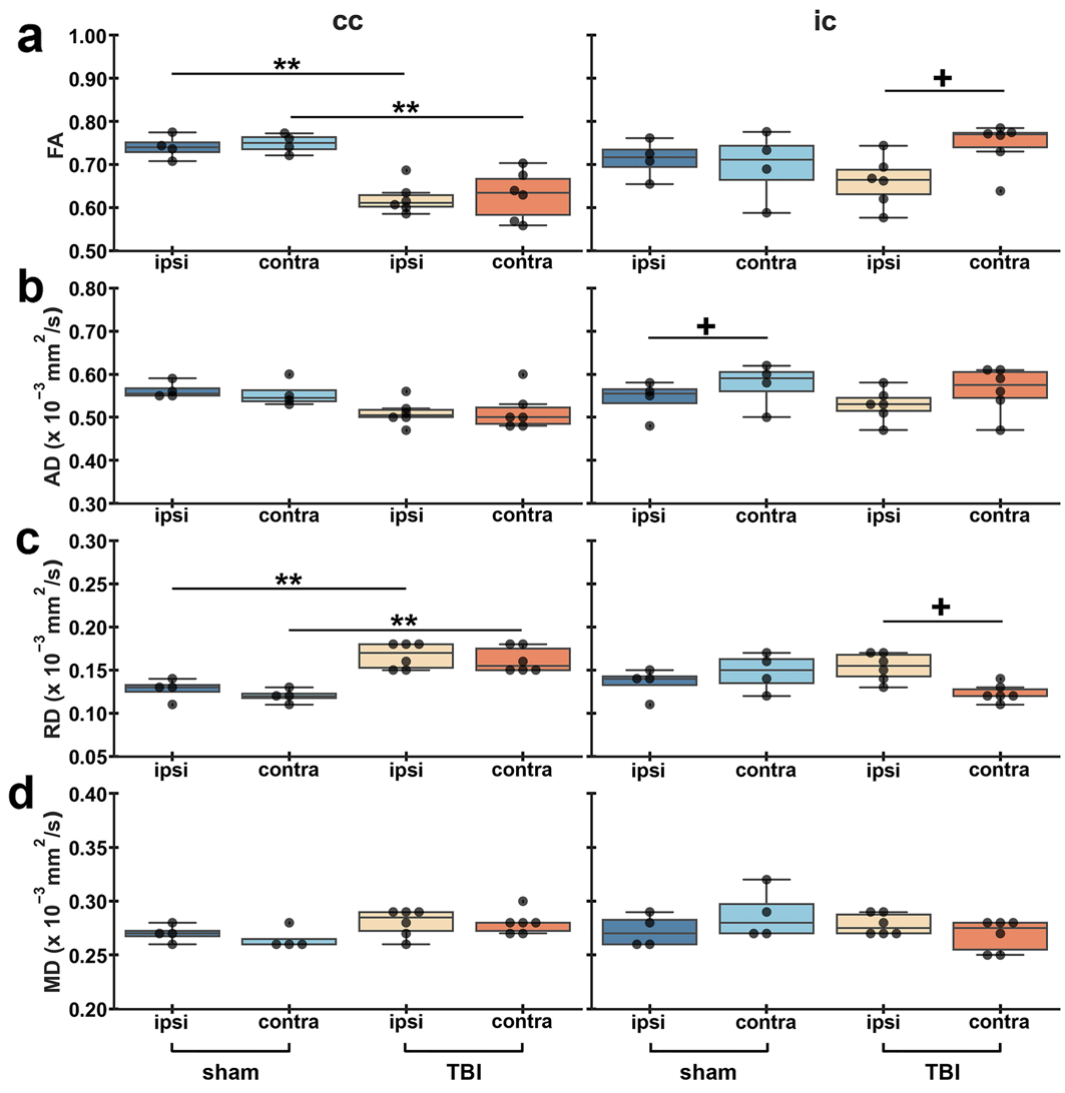
ROI-based analysis of tensor-based metrics in white matter areas. Results are shown as minimum, lower quartile, median, upper quartile and maximum, and paired t-test comparing ipsi- and contralateral sides within animals (**q < 0.01) or unpaired t-test comparing the same hemisphere between sham-operated and TBI rats (^+^*q* < 0.05) which are FDR corrected. Abbreviations: AD, axial diffusivity; cc; corpus callosum; FA, fractional anisotropy; ic; internal capsule; MD, mean diffusivity; RD, radial diffusivity.

The corpus callosum showed a significant decrease in FA ipsi- (*q* = 0.002) and contralaterally (*q* = 0.008) (Fig. 2a) and a significant increase in RD both ipsi- (*q* = 0.005) and contralaterally (*q* = 0.004) (Fig. 2c) in TBI rats when compared to sham-operated ones. In the internal capsule, TBI rats exhibited a significant decrease in FA (*q* = 0.015) (Fig. 2a) and a significant increase in RD (*q* = 0.028) ipsilaterally with respect to the contralateral side (Fig. 2c). Also, we found that the sham-operated rats exhibited a significant decrease in AD ipsilaterally (*q* = 0.026) as compared to the contralateral hemisphere (Fig. 2b). No significant differences were found in MD in those two areas (Fig. 2d).

**Figure 3:**
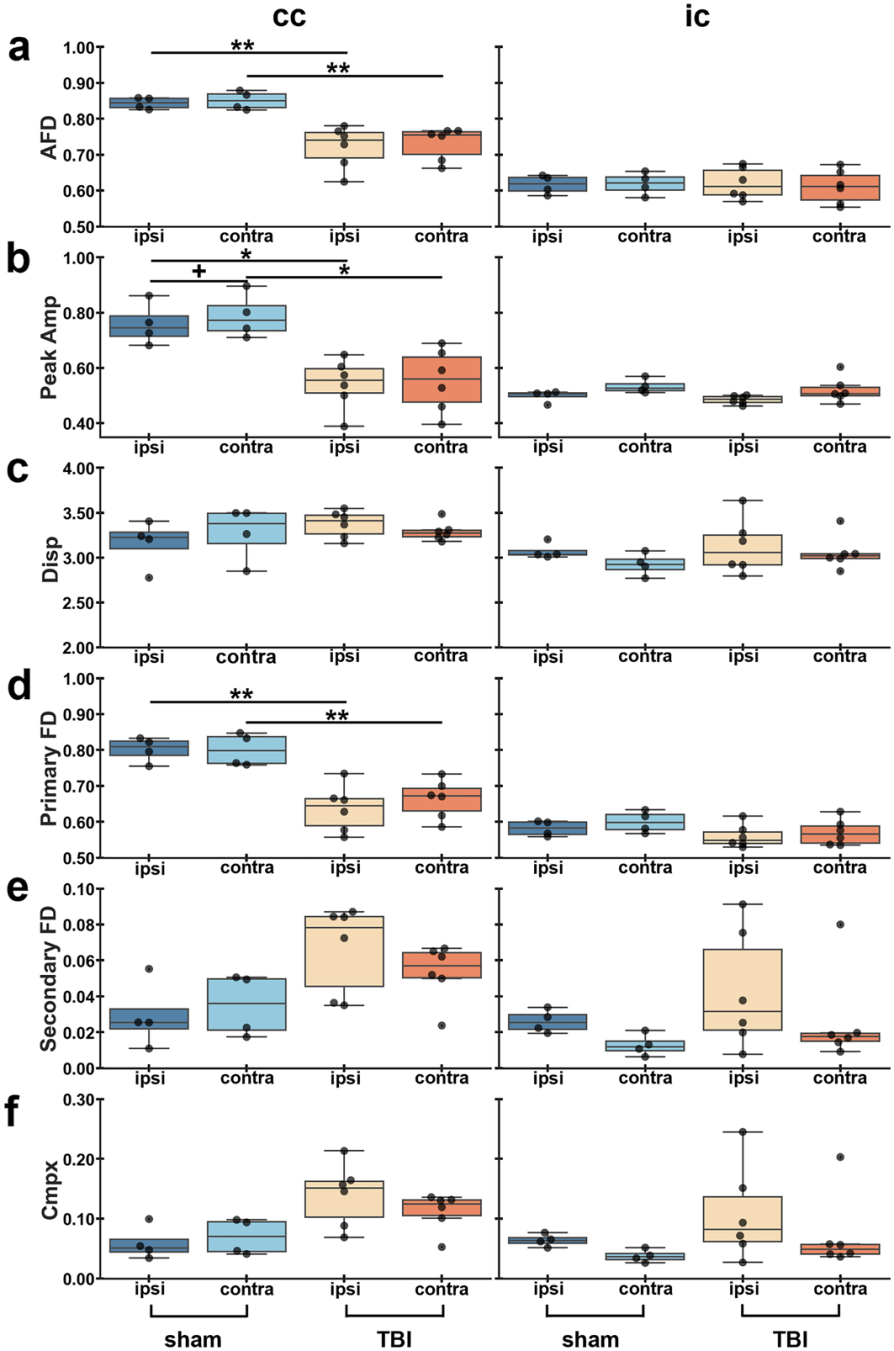
ROI-based analysis of fixel-based metrics in white matter areas. Results are shown as minimum, lower quartile, median, upper quartile and maximum, and paired t-test comparing ipsi- and contralateral sides within animals (*q < 0.05, **q < 0.01) or unpaired t-test comparing the same hemisphere between sham-operated and TBI rats (^+^*q* < 0.05) which are FDR corrected. Abbreviations: AFD, apparent fiber density; Amp, amplitude; cc; corpus callosum; Cmpx, complexity; Disp, dispersion; FD, fiber density; ic; internal capsule.

Fixel-based parameters revealed a significant decrease in AFD in the corpus callosum in TBI rats both ipsi- (*q* = 0.008) and contralaterally (*q* = 0.006) when compared to sham-operated rats (Fig. 3a). Peak FOD amplitude also showed a significant decrease ipsi- (*q* = 0.010) and contralaterally (*q* = 0.010) in TBI rats when compared to sham-operated, and a significant decrease ipsilaterally (*q* = 0.010) in sham-operated rats when compared to the contralateral side (Fig. 3b). For fiber bundle density metrics, we found a significant decrease in primary fiber bundle density in TBI rats ipsi- (*q* = 0.006) and contralaterally (*q* = 0.006) in the corpus callosum when compared to sham-operated rats (Fig. 3d). We observed a trend in secondary fiber bundle density and complexity in TBI rats (Fig. 3e and 3f). No significant differences were found in the internal capsule using fixel-based approach (Fig. 3).

**Figure 4:**
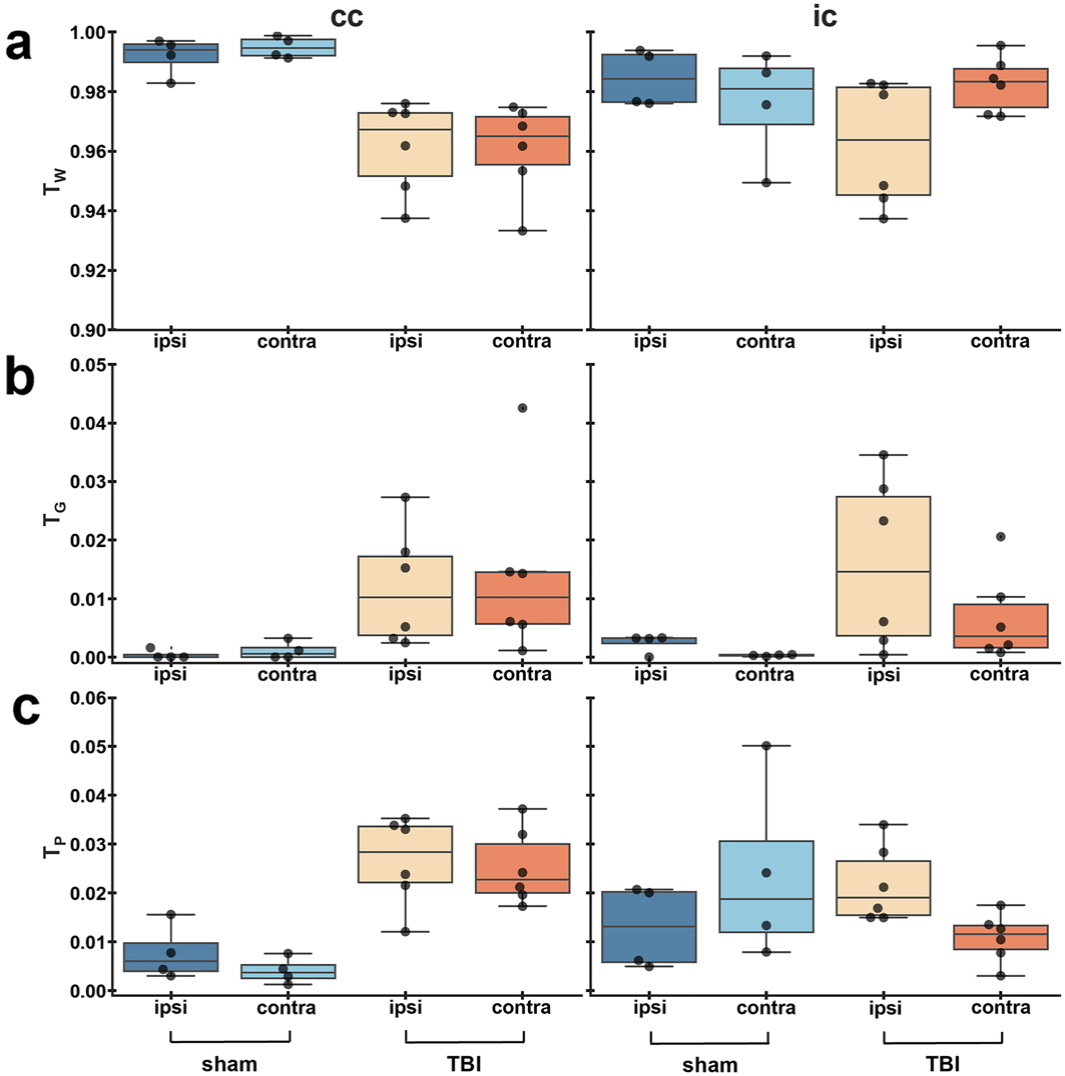
Relative 3-tissue signal fractions in white matter areas. Results are shown as minimum, lower quartile, median, upper quartile and maximum. Results of the compositional analysis are shown in Table 1. Abbreviations: cc; corpus callosum; ic, internal capsule; T_W_, T_G_ and T_P_, signal fraction maps representative of white matter, grey matter, and PBS, respectively.

The corpus callosum exhibited lower T_W_ (Fig. 4a), and markedly higher T_G_ (Fig. 4b), and T_P_ (Fig. 4c) ipsi- and contralaterally in TBI rats when compared to sham-operated. Statistical analysis of the isometric log-ratio transformed data showed a significant difference between TBI and sham-operated rats ipsilaterally (F(2, 7) = 10.735, Pillai’s trace = 0.754, *q* = 0.015) and contralaterally (F(2, 7) = 15.404, Pillai’s trace = 0.815, *q* = 0.011) (Table 1). The internal capsule exhibited lower T_W_, and higher T_G_ ipsilaterally in TBI rats when compared to sham-operated rats (Fig. 4). We obtained a significant difference contralaterally in TBI rats as compared to sham-operated (F(2, 7) = 11.516, Pillai’s trace = 0.767, *q* = 0.024) (Table 1).

**Table 1:** MANOVA of the isometric log-ratio transformed 3-tissue signal fractions (*ilr*_*1*_, *ilr*_*2*_).

Area	Test statistic	ipsi (sham) vs ipsi (TBI)	contra (sham) vs contra (TBI)	ipsi (sham) vs contra (sham)	ipsi (TBI) vs contra (TBI)
**cc**	**Pillai’s trace**	0.754	0.815	0.620	0.002
	**Num DF, Den DF**	2.000, 7.000	2.000, 7.000	2.0000, 5.000	2.0000, 9.000
	**F Value**	10.735	15.404	4.074	0.008
	**q-value**	0.015*	0.011*	0.119	0.992
**ic**	**Pillai’s trace**	0.338	0.767	0.324	0.423
	**Num DF, Den DF**	2.000, 7.000	2.000, 7.000	2.000, 5.000	2.000, 9.000
	**F Value**	1.787	11.516	1.196	3.297
	**q-value**	0.315	0.024*	0.376	0.169
**Cx**	**Pillai’s trace**	0.014	0.018	0.434	0.241
	**Num DF, Den DF**	2.000, 7.000	2.000, 7.000	2.000, 5.000	2.000, 9.000
	**F Value**	0.051	0.063	1.919	1.432
	**q-value**	0.950	0.950	0.577	0.577
**VB**	**Pillai’s trace**	0.734	0.242	0.914	0.407
	**Num DF, Den DF**	2.000, 7.000	2.000, 7.000	2.000, 5.000	2.000,9.000
	**F Value**	9.652	1.120	26.393	3.094
	**q-value**	0.019*	0.379	0.009**	0.127
**DG**	**Pillai’s trace**	0.386	0.277	0.260	0.281
	**Num DF, Den DF**	2.000, 7.000	2.000, 7.000	2.000, 5.000	2.000, 9.000
	**F Value**	2.199	1.339	0.877	1.763
	**q-value**	0.429	0.429	0.472	0.429
**l-m**	**Pillai’s trace**	0.993	0.655	0.024	0.734
	**Num DF, Den DF**	2.000, 7.000	2.000, 7.000	2.000, 5.000	2.000, 9.000
	**F Value**	0.700	6.654	0.061	12.430
	**q-value**	0.704	0.048*	0.942	0.010*

Bold text indicates statistically significant differences: *q_*(ilr1, ilr2)*_ < 0.05, **q_*(irl1, ilr2)*_ < 0.01. Abbreviations: cc, corpus callosum; Cx, perilesional cortex; Den, Denominator; DF, degrees of freedom; DG, dentate gyrus; ic, internal capsule; l-m, stratum lacunosum-molecure; Num, numerator; VB, ventrobasal complex.

**Figure 5:**
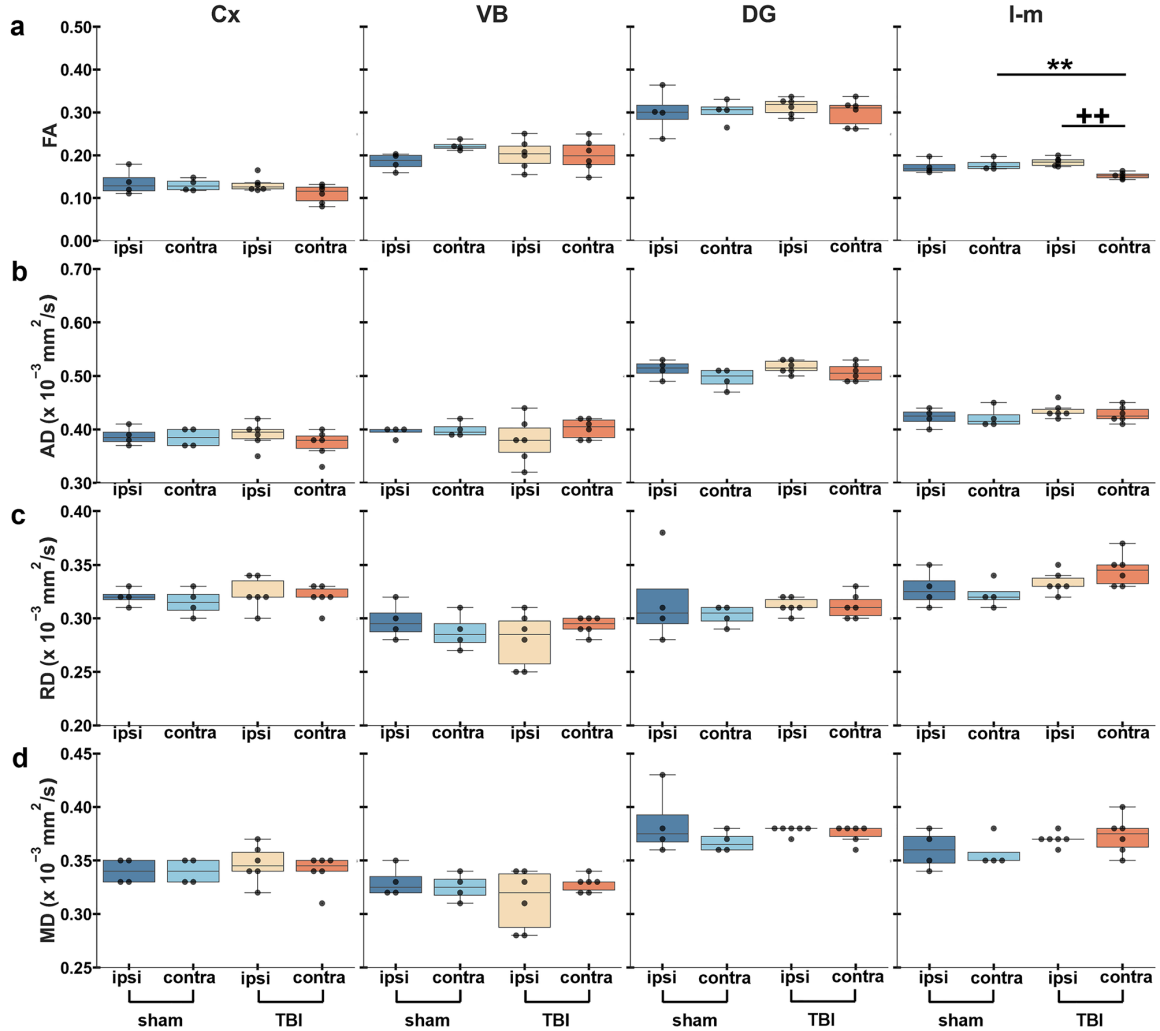
ROI-based analysis of tensor-based metrics in grey matter areas. Results are shown as minimum, lower quartile, median, upper quartile and maximum, and paired t-test comparing ipsi- and contralateral sides within animals (**q < 0.01) or unpaired t-test comparing the same hemisphere between sham-operated and TBI rats (^++^*q* < 0.01) which are FDR corrected. Abbreviations: AD, axial diffusivity; Cx; perilesional cortex; DG, dentate gyrus; FA, fractional anisotropy; l-m, stratum lacunosum-moleculare; MD, mean diffusivity; RD, radial diffusivity; VB, ventrobasal complex.

**Figure 6:**
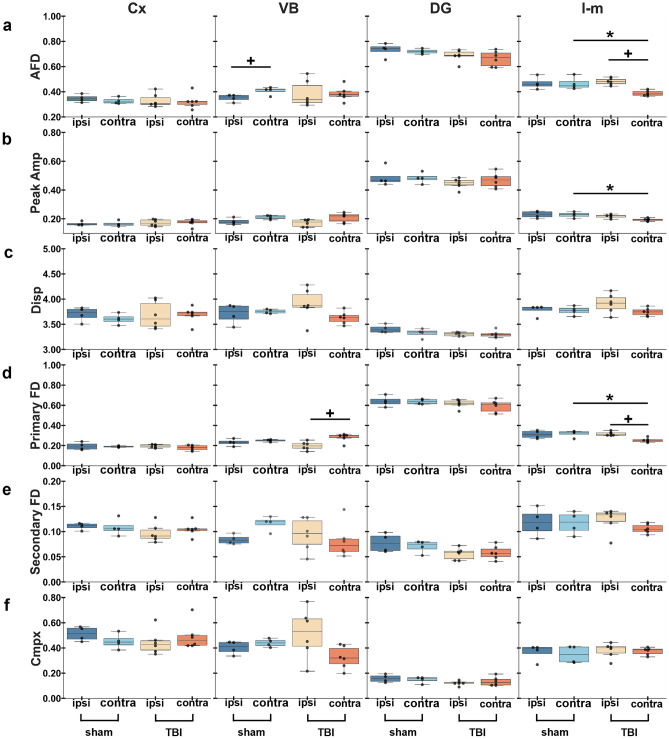
ROI-based analysis of fixel-based metrics in grey matter areas. Results are shown as minimum, lower quartile, median, upper quartile and maximum, and paired t-test comparing ipsi- and contralateral sides within animals (*q < 0.05) or unpaired t-test comparing the same hemisphere between sham-operated and TBI rats (^+^*q* < 0.05) which are FDR corrected. Abbreviations: AFD, apparent fiber density; Amp, amplitude; Cx, perilesional cortex; Cmpx, complexity; DG, dentate gyrus; Disp, dispersion; FD, fiber density; l-m, stratum lacunosum-moleculare; VB, ventrobasal complex.

In grey matter areas, DTI parameters only showed a significant decrease in the stratum lacunosum-moleculare in FA contralaterally (*q* = 0.009) in TBI rats when compared to the ipsilateral side, and when compared contralaterally to sham-operated rats (*q* = 0.008) (Fig. 5a). However, fixel-based parameters revealed more significant differences than DTI parameters (Fig. 5 and 6). The ventrobasal complex showed a significant increase in AFD ipsilaterally (*q* = 0.010) in sham-operated rats as compared to the contralateral side (Fig. 6a), and a significant decrease in primary fiber bundle density in TBI rats ipsi- and contralaterally (*q* = 0.012) (Fig. 6d). In the stratum lacunosum-moleculare, TBI rats showed a significant decrease in AFD contralaterally when compared to the ipsilateral side (*q* = 0.014) and when compared to the contralateral side in sham-operated rats (*q* = 0.017) (Fig. 6a), and a significant decrease in peak FOD amplitude contralaterally (*q* = 0.030) in TBI rats when compared to sham-operated rats (Fig. 6b). Also in this area, we found a significant decrease primary fiber bundle density contralaterally in TBI rats when compared to sham-operated rats (*q* = 0.011) and a significant increase in TBI rats ipsilaterally (*q* = 0.011) as compared to the contralateral side (Fig. 6d).

**Figure 7:**
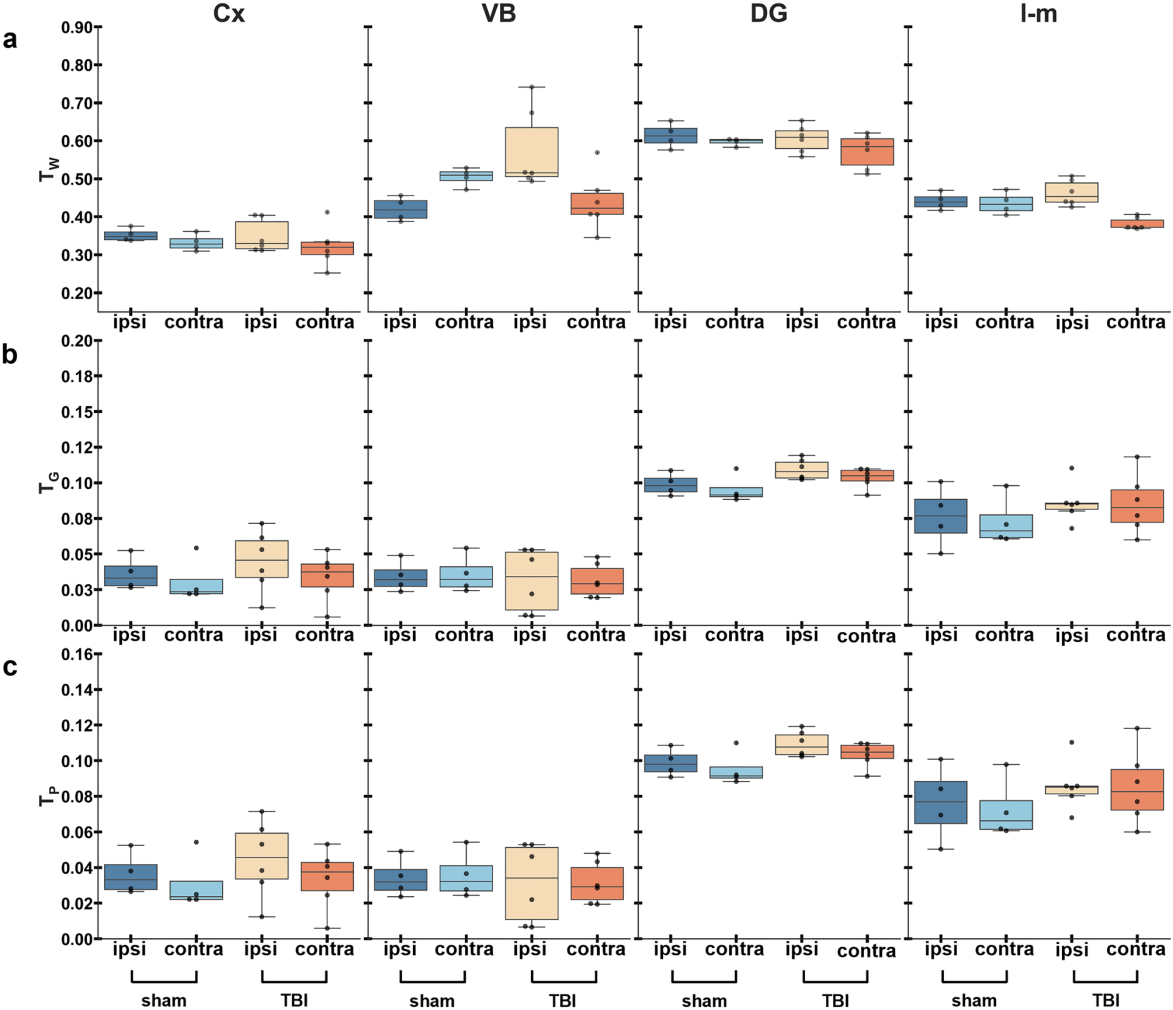
Relative three-tissue signal fractions in grey matter areas. Results are shown as minimum, lower quartile, median, upper quartile and maximum. Results of the compositional analysis are shown in Table 1. Abbreviations: Cx, perilesional cortex; DG, dentate gyrus; T_W_, T_G_, T_P_, signal fraction maps representative of white matter, grey matter, and PBS; l-m, stratum lacunosum-moleculare; VB, ventrobasal complex.

We observed higher T_W_ ipsilaterally in TBI rats when compared to sham-operated in the ventrobasal complex (Fig. 7). Statistical analysis of the isometric log-ratio transformed data revealed a significant difference ipsilaterally in the ventrobasal complex between sham-operated and TBI rats (F(2, 7) = 9.652, Pillai’s trace = 0.734, *q* = 0.019), and ipsi-contralaterally in sham-operated rats (F(2, 5) = 26.393, Pillai’s trace = 0.914, *q* = 0.009) (Table 1). In the stratum lacunosum-moleculare, we found higher T_W_ ipsilaterally when compared to contralateral hemisphere (Fig. 7). The statistical analysis showed a significant difference contralaterally between sham-operated and TBI rats (F(2, 7) = 6.654, Pillai’s trace = 0.655, *q* = 0.048), and ipsilaterally as compared to the contralateral hemisphere in TBI rats (F(2, 9) = 12.430, Pillai’s trace = 0.734, *q* = 0.010) (Table 1).

**Figure 8:**
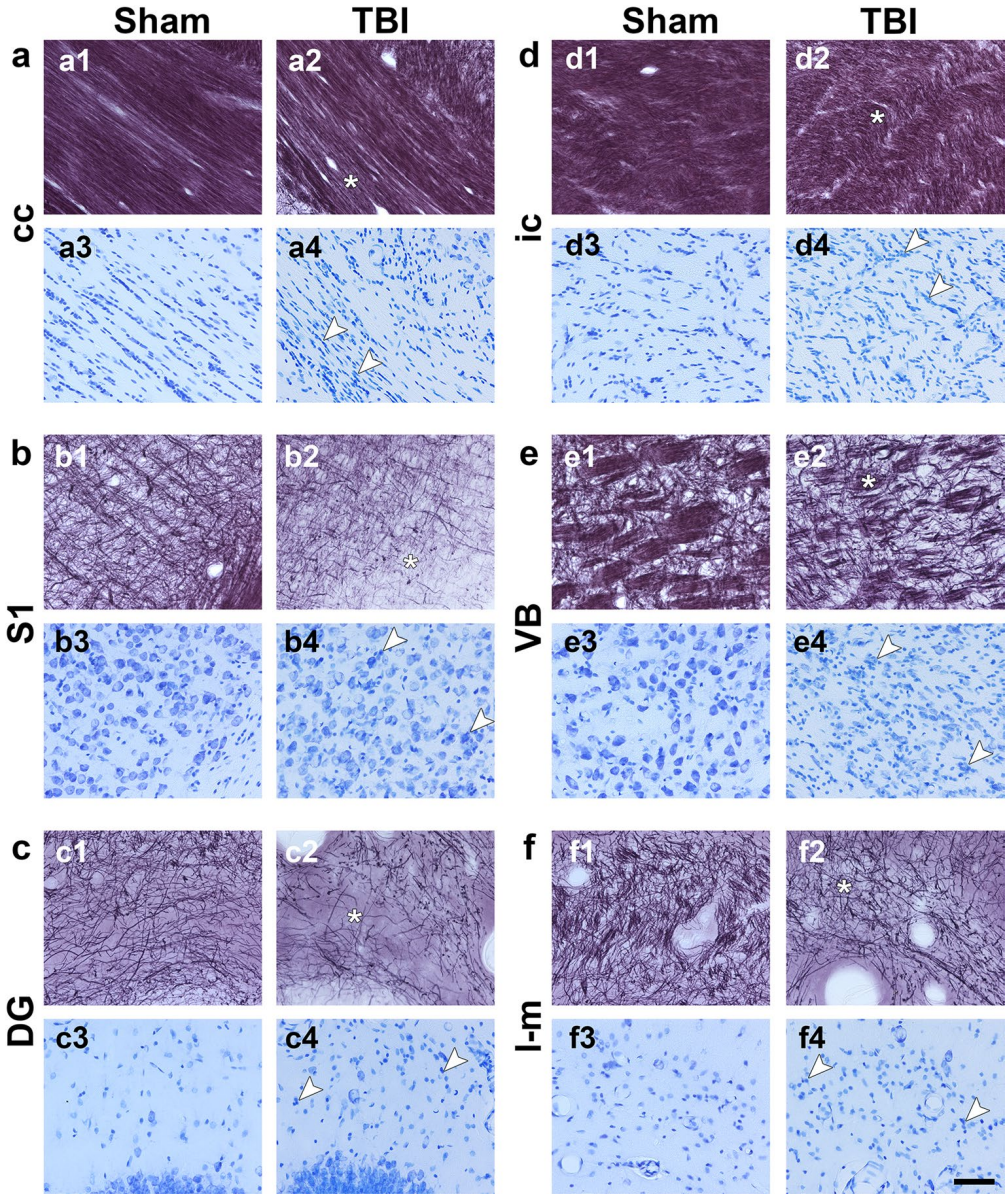
Representative photomicrographs of Nissl and myelin stainings from a sham-operated and a TBI rat. Asterisks indicate a decrease in myelin density and arrowheads increased cell density (small and intense blue cells) associated to gliosis. Abbreviations: cc, corpus callosum; DG, dentate gyrus; ic, internal capsule; l-m, stratum lacunosum-moleculare; S1, primary somatosensory cortex; VB, ventrobasal complex. Scale bar: 50 µm.

Figure 8 shows representative animals from both sham-operated and TBI groups. We observed loss of myelinated axons and increased cellularity in all the brain areas included in this study. Myelin-stained sections showed a sparse loss of myelin in the deeper aspects of the corpus callosum (Fig. 8a2) and dorsally in the internal capsule (Fig. 8d2) accompanied by the presence of gliosis along the structures as seen in Nissl staining (Fig. 8a4 and 8d4). In grey matter areas, we found extensive loss of myelinated axons and gliosis in the somatosensory cortex (Fig. 8b2 and 8b4) and stratum lacunosum-moleculare (Fig. 8f2 and 8f4), and less severe in the molecular layer of the dentate gyrus (Fig. 8c2 and 8c4). The ventrobasal complex showed the thinning of fiber bundles across these nuclei, and extensive neuronal loss and gliosis (Fig. 8e2 and 8e4).

**Figure 9:**
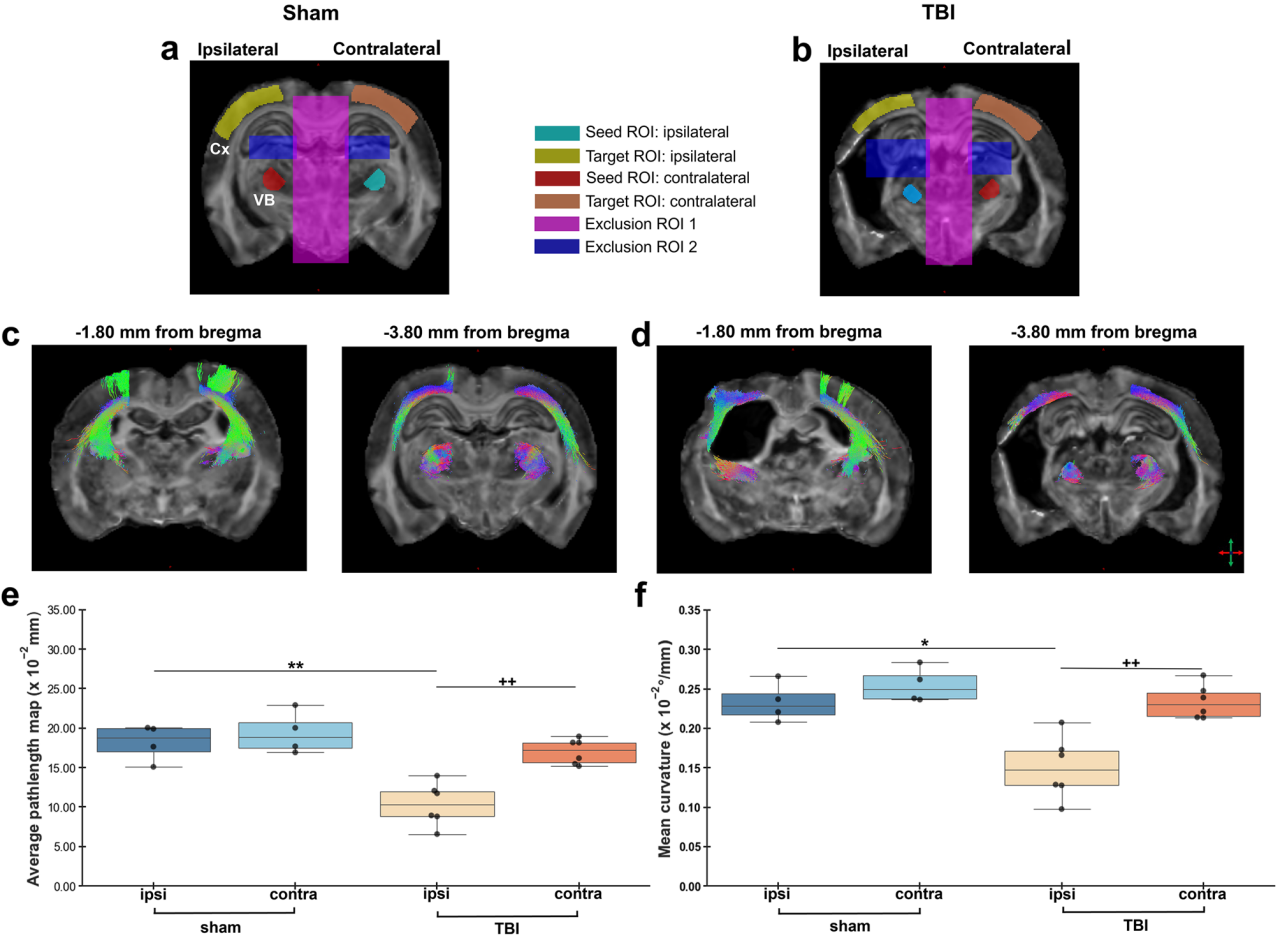
Targeted tractography. Probabilistic streamlines were generated using a combination of seed, target, and exclusion ROIs in sham-operated (**a**) and TBI (**b**) rats. 2D reconstruction of the thalamocortical pathway in a representative sham and TBI rat at − 1.80 mm and − 3.80 mm from bregma (c, d), colours indicate directionality: red, medio-lateral; green, dorso-ventral; blue, rostro-caudal. Track-weighted measures of average pathlength map and mean curvature computed from the generated tractograms (e, f). Results are shown as minimum, lower quartile, median, upper quartile and maximum, and paired t-test comparing ipsi- and contralateral sides within animals (*q < 0.05, **q < 0.01) or unpaired t-test comparing the same hemisphere between sham-operated and TBI rats (^++^*q* < 0.01) which are FDR corrected. Abbreviations: Cx, perilesional cortex; VB, ventrobasal complex.

We segregated the thalamocortical pathway with TWI for further analysis. Figure 9 shows the seed, target, and exclusion ROIs used to generate the thalamocortical pathway in the ipsi- and contralateral hemispheres of a representative sham-operated (Fig. 9a) and TBI (Fig. 9b) rats. Figure 9 shows the probabilistic streamlines generated along the thalamocortical pathway distant to the lesion site at − 1.80 mm from bregma, and close to the lesion site at − 3.80 mm (Fig. 9c and 9d). Visually, at − 3.80 mm from bregma, streamlines lining the external capsule ipsilaterally in TBI rats exhibited changes in orientation from dorso-ventral to rostro-caudal (Fig. 9c and 9d). Distant from the lesion site, at − 1.80 mm, streamlines emanating from the thalamus into the perilesional cortex showed more prominent changes in orientation from dorso-ventral to rostro-caudal ipsilaterally in TBI rats as compared to sham-operated (Fig. 9c and 9d). In TWI metrics, we found a significant decrease in average pathlength and curvature ipsilaterally (*q* = 0.004, *q* = 0.012) in TBI rats as compared to sham, and in TBI rats ipsi-contralaterally (*q* = 0.004, *q* = 0.008) (Fig. 9e and 9f).

## Discussion

TBI provokes a myriad of secondary microstructural changes in the brain, which can last months and years in patients after the injury. Therefore, we used conventional and advanced diffusion MRI measures to enable more comprehensive detection of microstructural differences in white and grey matter areas in a rat model of severe TBI at a chronic time point. Tensor-based measures were sensitive to changes in the corpus callosum, internal capsule and stratum lacunosum-moleculare while fixel-based measures detected changes only in the corpus callosum and stratum lacunosum-moleculare. Compositional analysis revealed distinct changes in 3-tissue signal fractions in the corpus callosum, internal capsule, ventrobasal complex and stratum lacunosum-moleculare. Additionally, TWI-based measures detected changes in morphology of the thalamocortical tract in TBI rats as compared to sham-operated ones. Myelin- and Nissl-stained sections revealed decreased myelin density and increased cellularity, indicating ongoing microstructural alterations in these brain areas five months after injury.

Our study illustrates that both tensor- and fixel-based approached have sensitivity to detect complex microstructural changes associated to severe TBI in both white and grey matter. Similarly, as reported by^57^ the splenium of the corpus callosum and internal capsule in TBI rats at the chronic phase showed decreased FA and AD, and/or increased RD related to microstructural alterations including loss of myelinated axons and/or presence of iron deposits. Additionally, we found that increased cell density in both the corpus callosum and internal capsule corresponds with increased RD observed in these areas. With regards to CSD metrics^30^, reported decreased AFD in the corpus callosum bilaterally, fimbria, external capsule, and corticospinal tract ipsilaterally in TBI rats. In our study, the observed reductions in AFD, peak FOD amplitudes in the corpus callosum are indicative of myelin loss, while the reduced primary FD could be associated to increased cell density in the area. Additionally, the corpus callosum in our work demonstrated lower T_W_ with markedly higher T_P_, and an increase in T_G_ ipsilaterally in TBI rats. The corpus callosum showed substantial myelin loss in the deeper aspects, which might lead to an increase in free diffusion leading to markedly higher T_P_, while the contribution of gliosis might explain the increase observed in T_G_. On the contrary, lower T_W_ and higher T_G_ observed in the internal capsule could be indicative of less severe myelin loss overall and gliosis. Although, there are no other reported studies in TBI to corroborate these microstructural changes observed in the 3-tissue signal fraction maps, previous studies utilizing SS3T-CSD have reported similar results when characterizing microstructural heterogeneity of white matter lesions in patients with Alzheimer’s disease^35^ or stroke^41^.

In grey matter, previous studies utilizing diffusion MRI to investigate severe TBI have demonstrated changes in the ventrobasal complex^57^ and stratum lacunosum-moleculare^24^. More specifically^57^, observed a significant decrease in RD in the ventrobasal complex ipsilaterally in TBI animals, related to thinning of fiber bundles, myelin loss, neurodegeneration, and presence of calcifications^12^. Our DTI metrics did not detect any change in the area, but we observed a significant decrease in primary FD and higher T_W_ ipsilaterally in TBI animals compared to sham-operated ones. The decreased FD could reflect the thinning of fiber bundles across the nuclei. However, higher T_W_ was not indicative of a higher number of myelinated axons, but it was rather associated with extensive gliosis and the presence of glial processes^68^. We also observed significant decreases in the primary fibre bundle density, and slightly elevated secondary fibre bundle density in the area. Previous studies^53,67^ have suggested that the loss in density of the white matter fiber bundles causes other secondary injury to fill this area. These processes restrict the isotropic diffusion signal in a manner that mimics the presence of crossing fibres, which manifests as an increase in the secondary FD^53,67^. The ventrobasal complex is a region with a mixture of multiple fiber bundles and grey matter. From our findings, we can deduce that Tw in such scenarios can be influenced from secondary processes, such as myelin loss or gliosis, and thereby requires careful interpretation^52,53,67^. In the stratum lacunosum-moleculare, our DTI measures revealed increased FA, and SS3T-CSD showed increased AFD, primary FD, and higher T_W_, and T_G_ ipsilaterally in TBI rats. The observed increases in all these metrics indicate an overall increase in anisotropy of the underlying tissue microstructure. Previously^24^, did not observe any change in FA ipsilaterally in TBI rats as compared to controls, but the authors revealed changes in the principal diffusion direction towards the mediolateral orientation, associated to substantial loss of myelinated axons and increased gliosis, which might explain the observed increase in diffusion anisotropy. In the dentate gyrus, we did not find changes in either conventional or advanced diffusion MRI measures. However, our histological assessment demonstrated less severe myelin loss and gliosis in the area as compared to other grey matter areas, while^24^ also observed mild mossy fiber sprouting and gliosis ipsilaterally as compared to sham-operated rats.

Our study did not reveal any changes in the somatosensory cortex, which was unexpected considering the proximity to the lesion site. However, when segregating the thalamocortical pathway, TWI metrics revealed significantly shorter streamlines and reduced curvature in TBI rats ipsilaterally when compared to sham-operated ones. We suspect the failure of our ROI-based approach to detect any changes in the area resulted from the combination of varying cortical atrophy following trauma, presence of crossing fibers, or associated microstructural alterations including substantial myelin loss, gliosis, or neurodegeneration in TBI animals. Using TWI^30^, reported streamlines that were fewer in number with shorter and straighter trajectories in the ipsilateral corpus callosum, internal capsule, and fimbria in TBI rats as compared to sham-operated. Moreover, these authors also demonstrated only connectivity-based fixel enhanced AFD and TWI measures to be sensitive to degeneration of the corticospinal tract following severe TBI as compared to voxel-based DTI measures. Therefore, we suspect that using a complete fixel-based approach^30,31,49,67,69^ as opposed to our ROI-based approach may have provided more sensitivity to our fixel-based measures to detect changes in the somatosensory cortex. Overall, our findings highlight the utility of combining several approaches when investigating the complex microstructural alterations following severe trauma at a chronic phase.

Diffusion MRI data, typically employing SE-EPI sequences are commonly characterized by low SNR and CNR, and affected by multiple artefacts^47^. In this regard, scanning rat brains ex vivo provided 3D isotropic data with high resolution and SNR, which facilitated reliable application of SS3T-CSD and tractography methods for assessing TBI-related microstructural complexity. Even though, our dMRI acquisition scheme is lower than recommended for the AFD framework^70,71^, previous studies have applied CSD with lower b-value datasets^72–74^. Furthermore, using a lower b-value and angular resolution dMRI scheme (1000 s/mm^2^, 30 directions), a previous study^75^ demonstrated the accuracy and interpretation of CSD measures to be sufficiently accurate when compared to results at 3000 s/mm^2^^70^. In addition, the authors found CSD-derived AFD to be provide more insights into the WM microstructure as compared to the tensor-based metrics similar to our findings, and also improved fiber tractography. These studies reinforce the biological meaningfulness of CSD-based metrics even at a lower b-value regime as in our study. Nevertheless, the *b-*value used may be insufficient to completely overcome the reduced apparent diffusion coefficient, observed throughout the brain ex vivo as reported previously^67,76–78^. Moreover, our acquisition protocols would also be tailored to include multiple shells that would enable us to incorporate typically used mathematical microstructural models such as neurite orientation and dispersion density imaging^79^. Future studies would also be planned in vivo with the possibility of including multiple time-points, allowing monitoring of the TBI-related tissue changes over time, which in-turn would enable a clearer understanding of the TBI pathology and improved clinical translatability^80^.

Previous studies utilizing both single- and multi-shell CSD have demonstrated to be more precise in detecting microstructural alterations in severe and mild TBI as compared to DTI^30,31,67,69^. We have used SS3T-CSD in our study as it allows to derive several quantitative measures including fixel-based, signal fraction maps, and TWI with a single-shell acquisition in a time efficient manner. This would also enable the incorporation of other quantitative MRI contrasts such as *T*_2_*, and quantitative susceptibility mapping to explore other tissue changes such as iron accumulation or calcifications following TBI^49,81,82^. Although, myelin loss was observed in several areas in the TBI animals, it must be highlighted that diffusion MRI is not directly sensitive to myelin^83,84^, and hence, decreased FD metric observed in our study does not necessarily imply myelin loss or demyelination, rather processes that accompany or follow changes in myelin post-trauma^83,84^. In addition, the use of SS3T-CSD has demonstrated to estimate WM FODs more reliably in presence of partial volume effects as compared to single-tissue CSD^39^, changes detected in average pathlengths can be also influenced by aspects other than actual pathlengths, such as gliosis. Previously by using the severe LFPI model, we have demonstrated astroglial and microglial activation in the ipsilateral thalamus at the chronic time-point^49^. In this study, we focused on the association of microstructural changes in the diffusion signal profiles. The beneficial or detrimental role of inflammatory cells in the injured brain at the chronic phase will be considered in future studies using specific histological markers. In addition, a single anisotropic component may be inadequate to represent cortical anisotropy^85^ and even different WM regions of the brain^86^. Future work should, therefore, investigate the use of multiple FODs as suggested by^85^ in a proof-of-concept study.

The experimental severe TBI animal model used in our study caused extensive deformation ipsilaterally in the brain. Therefore, we resorted to voxel-wise analysis of the FBA metrics, as co-registration of the individual brains was unfeasible. Thus, we could not completely utilize the specificity of individual fixels provided by FBA in our study as used previously^30,31,67,69^. We also observed differences between ipsi- and contralateral hemispheres in sham-operated rats, which could result from the effects of the sham-operation also reported previously^12,24,30,31,57,67,69,87,88^. In this regard, future studies would also benefit from the inclusion of naïve animals to overcome this limitation. Moreover, future studies would also benefit with the inclusion of both males and females to better investigate the influence of gender-related differences post-trauma^89–91^.Regarding tractography, we observed most prominent changes distal from the lesion at − 1.80 mm bregma, compared to the lesion site at − 3.80 mm, because we limited our tract selection and radius of curvature to 10,000 and 45 degrees respectively. Future studies exploring the variation in streamline tractography parameters will be useful to enhance the assessment of track-related changes following trauma. Finally, it would be beneficial to corroborate the quantitative MRI and tractography findings with quantitative histology^68,92^ or tract-tracing^93^. As this is a first preclinical study characterizing SS3T-CSD with histology, the advantages/limitations from our findings can potentially serve as a reference to investigate tissue-specific changes in other experimental and human pathological studies in the brain.

In conclusion, this study demonstrates the potential of conventional and advanced diffusion MRI measures for investigating microstructure-related tissue changes following severe TBI at a chronic stage. The combination of DTI, and 3-tissue CSD derived fixel-based, TWI, and compositional analysis of the signal fraction maps provide further insights into the complex nature of these microstructural changes in both white and grey matter areas. Histological corroboration of these findings can further improve our understanding of the underlying pathophysiology and reinforce the use of combining conventional and advanced measures to investigate the chronic outcomes following severe injury.

## Supplementary Information

Supplementary Figure S1.

## Data Availability

The data from this study are available upon request from the corresponding author.

## Abbreviations

AFD: Apparent fiber density
AD: Axial diffusivity
CSD: Constrained spherical deconvolution
CSF: Cerebrospinal fluid
DTI: Diffusion tensor imaging
FA: Fractional anisotropy
FBA: Fixel-based analysis
FODs: Fiber orientation distributions
GM: Grey matter
LFP: Lateral fluid percussion
MRI: Magnetic resonance imaging
PBS: Phosphate-buffered saline
RD: Radial diffusivity
ROIs: Regions-of-interest
SE-EPI: Spin-echo-echo-planar-imaging
SS3T-CSD: Single-shell 3-tissue CSD
TBI: Traumatic brain injury
TWI: Track-weighted imaging
