# Supplementary material for: Diffusion MRI approaches for investigating microstructural complexity in a rat model of traumatic brain injury
Source: Sci Rep. 2023 Feb 8;13:2219. doi: 10.1038/s41598-023-29010-3 (PMC9908904; doi:10.1038/s41598-023-29010-3)
Supplement: Supplementary file 1 — Supplementary Figure S1. [file 41598_2023_29010_MOESM1_ESM.docx]

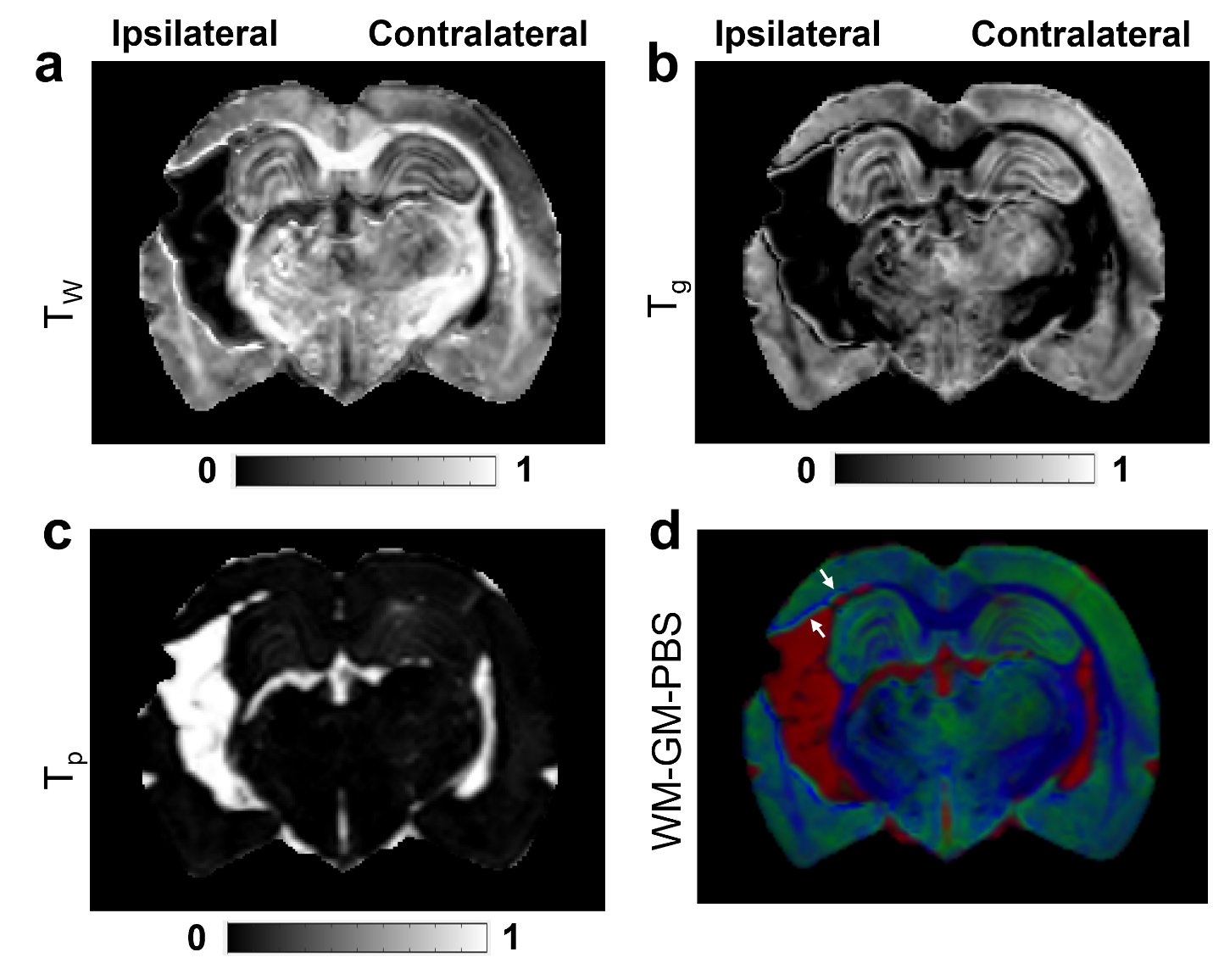


**Supplementary Figure 1.** SS3T-CSD effectively decomposes the different signal contributions to enable better representation of the pathological tissue. 3-tissue signal fraction maps (a-d) in a representative TBI rat. Colors represent tissue types: blue - WM; green - GM; red - PBS. For example, areas affected by TBI close to the ventricles (shown by white arrows) might be less influenced by the effects of partial volume by SS3T-CSD.
